# Risk factors to cause tooth formation anomalies in chemotherapy of paediatric cancers

**DOI:** 10.1111/ecc.12038

**Published:** 2013-01-21

**Authors:** S Nishimura, H Inada, Y Sawa, H Ishikawa

**Affiliations:** Department of Oral Growth & Development, Fukuoka Dental CollegeSawara-ku, Fukuoka, Japan; Department of Pediatrics and Child Health, Kurume University School of MedicineSawara-ku, Kurume; Department of Morphological Biology, Fukuoka Dental CollegeSawara-ku, Fukuoka

**Keywords:** growth and development, medical disability, orthodontics, tooth formation anomalies, anti-cancer chemotherapy

## Abstract

This study aimed to investigate the risk factors of tooth formation anomalies in anti-cancer chemotherapies. Long-term survivors treated by conventional chemotherapy (*n* = 26), conventional chemotherapy with high-dose chemotherapy (HDC) (*n* = 14), and HDC with total body irradiation (TBI) (*n* = 6) were analysed for the incidence of tooth agenesis, microdonts, and short-rooted teeth. The tooth agenesis and/or microdonts were found in second premolars and second molars, but not in first molars or central incisors. The ratio of subjects with tooth agenesis and/or microdonts was 66.7% and 18.2% in subjects administered conventional chemotherapy at <4 years and ≥4 years of age, respectively, while it was 100% and 25% in subjects administered HDC at <4 years and ≥4 years of age. The incidence of tooth formation anomalies did not related with the duration of conventional chemotherapy but increased by HDC. The incidence of tooth formation anomalies did not show significantly differences between the HDC with and without TBI groups, and was higher in busulfan-administered subjects than in subjects given cyclophosphamide. It may be concluded that the high-risk group of tooth agenesis is the subjects with HDC under 4 years of age. However, protocols of conventional chemotherapy are not an important risk factor to cause the tooth formation anomalies.

## Introduction

A radical cure for paediatric cancers, high-dose chemotherapy using alkylators (HDC) accompanied with the transplantation of haematopoietic stem cells derived from bone marrow or peripheral blood is in use today. The HDC and total body radiation (TBI) have improved the rate of cured paediatric cancers considerably than conventional chemotherapies but given rise to problems for the later quality of life (QOL) in long-term survivors. Among paediatricians, it has been known that tooth formation anomalies occur in persons who have been subjected to chemotherapy or radiotherapy in childhood, at ages corresponding to the permanent tooth formation period. In the 1980s–1990s, anticancer drug or radiotherapy-induced tooth formation anomalies came to be reported, suggesting that anticancer therapies cause irreversible morphological changes in permanent teeth: tooth agenesis, microdonts, and short-rooted teeth, which have been ascribed to arrested development of crowns and roots of permanent teeth (Maguire *et al*. 1987; Dahllof *et al*. 1988; Nasman *et al*. 1997; Uderzo *et al*. 1997; Kaste *et al*. 1998; Minicucci *et al*. 2003; Oguz *et al*. 2004; Cubukcu *et al*. 2012). In 2002, it has been reported that HDC is an important risk factor causing tooth formation anomalies and that chemotherapy-induced tooth formation anomalies emerge in the first and second premolars, and the second molars in children (Hölttä *et al*. 2002, 2005a,b; van der Pas-van Voskuilen *et al*. 2009). Further, it has been reported that TBI and high-dose alkylators act as independent risk factors (Duggal 2003; Kaste *et al*. 2009; Hsieh *et al*. 2011). Overall, it appears that the main factor in tooth formation anomalies is the age when chemotherapy is administered but further study of chemotherapy-induced tooth formation anomalies is still required to establish dental guidelines for anti-cancer treatments with not only HDC and also conventional chemotherapy, as the available data are insufficient because of the presently short history of long-term survivors who have undergone HDC, covering only about 20 years.

This study aimed to investigate the kind of teeth suffering from chemotherapy-induced anomalies, and analysed the relation of tooth formation anomalies to the risk factors: the age at chemotherapy, chemotherapy duration, kinds of alkylators, and TBI. It is thought that there is an urgent need for an immediate accumulation of studies of HDC-induced tooth formation anomalies to suggest dental guidelines for anti-cancer care.

## Subjects and Methods

### Subjects and treatment protocols

The examination was conducted for persons willing to take part in this study at the Kurume University Hospital (Kurume, Japan) and at the Fukuoka Dental College Medical and Dental Hospital (Fukuoka, Japan), and informed consent was obtained from all participants and/or parents. The protocol was reviewed and approved by the Institutional Review Board of both the Kurume University Hospital (Kurume, Japan) and the Fukuoka Dental College Medical and Dental Hospital (Fukuoka, Japan) in conformation with the principles of the Helsinki Declaration.

The oral examination was conducted on 46 long-term survivors treated for paediatric cancers (Table 1) with an average age of 17.7 years. The subjects were divided into two groups: a conventional chemotherapy group of 26 subjects (12 boys, 14 girls) who had undergone only conventional chemotherapy; a conventional chemotherapy and HDC group of 20 subjects (15 boys, 5 girls) who had undergone HDC after the conventional chemotherapy, and followed by autologous peripheral blood stem cell transplantation (*n* = 12), allogenic peripheral blood stem cell transplantation (*n* = 1), or allogenic bone marrow transplantation (*n* = 7), according to the individually modified treatment protocols of the Kurume University Hospital between 1983 and 2008 when the subjects were under 10 years of age (onset ages 0.0–9.8 years; mean 3.7) (Table 1, Table S1). In multi-agent HDC using alkylators, busulfan (Bus) and cyclophosphamide (Cyc) were the most frequently used (Table 2). The durations of the chemotherapy are 0.3–4.0 years (mean 2.1) for the conventional chemotherapy group and 0.3–3.6 (mean 1.3) years for the HDC group. Some subjects have undergone fractionated HDC for 2–4 days. There were 17 children who received cranial, craniospinal and focal irradiations. These irradiations do not affect tooth development because the face is fully protected by radiation shades. There are also six children who received TBI (Table 1). In consideration of the influence of TBI, the HDC group was subdivided into groups with TBI (Table S1).

**Table 1:** Diagnosis and treatment contents

		CC		CC + HDC								
			
Numbers of subjects			(+IR)		Alkylators in the multi-agent HDC						(+IR)	
					
Diagnosis	Total	Subtotal	CRI/CSI/FI	Subtotal	Bus	Cyc	Mel	Tio	Ran	Ifo	TBI	CRI/CSI/FI
ALL	26	19	8	7	3	6	1	0	1	0	5	4
AML	6	2	0	4	4	1	3	0	0	0	0	0
MBL	4	1	1	3	1	2	1	2	1	0	0	3
PNET	2	0	0	2	1	0	1	1	0	0	0	1
NBL	2	1	0	1	1	1	1	1	0	1	1	1
HBL	2	1	0	1	0	0	1	1	0	0	0	0
SB	1	0	0	1	1	0	1	0	0	0	0	0
ACC	1	1	0	0	0	0	0	0	0	0	0	0
ML	1	1	1	0	0	0	0	0	0	0	0	0
WT	1	0	0	1	0	1	0	1	0	0	0	1
Total	46	26	10	20*	11	11	9	6	2	1	6†	10‡

* Seven subjects without IR.

† Three subjects with TBI (12 Gy) only.

‡ Seven subjects with CRI/CSI/FI only.

†,‡Three subjects with TBI and CRI/CSI/FI.

Single dose values of alkylators as follows: busulfan (Bus) 448–576 mg/m^2^, cyclophosphamide (Cyc) 1200–4500 mg/m^2^, melphalan (Mel) 180–540 mg/m^2^, thiotepa (Tio) 600–900 mg/m^2^, ranimustine (Ran) 250–450 mg/m^2^, ifosfamide (Ifo) 480 g/m^2^.

ACC, adrenal cortical carcinoma; ALL, acute lymphoblastic leukemia; AML, acute myeloid leukemia; CC, conventional chemotherapy; CRI, cranial irradiation; CSI, craniospinal irradiation; FI, focal irradiation; HBL, hepatoblastoma; HDC, high-dose chemotherapy; IR, irradiation; MBL, medullo blastoma; NBL, neuroblastoma; NHL, non-Hodgkin's lymphoma; PNET, primitive neuroectodermal tumour; SB, undifferentiated sarcoma of bone; WT, Wilm's tumour.

**Table 2:** Incidence of permanent teeth with formation anomalies

Study group		TA (%)	MO (%)	SR (%)	TA or MO (%)	TA, MO, or SR (%)	TFA scores
CC (*n* = 26)	Range	0.0–7.1	0.0–28.6	0.0–57.1	0.0–28.6	0.0–64.3	0.0–22.0
	Average	0.3	5.8	19.0	6.0	25.0	8.8
CC + HDC without TBI (*n* = 14)	Range	0.0–39.3	0.0–14.3	0.0–78.6	0.0–50.0	0.0–100	0.0–49
	Average	8.7	4.9	49.7	13.5	63.3	23.9
CC + HDC with TBI (*n* = 6)	Range	0.0–28.6	0.0–21.4	28.6–78.6	0.0–50	28.6–100	8.0–50
	Average	6.0	8.9	54.8	14.9	69.6	25.3
Total (*n* = 46)	Range	0.0–39.3	0.0–28.6	0.0–78.6	0.0–50.0	0.0–100.0	0.0–50
	Average	3.6	5.9	33.0	9.5	42.5	15.5

CC, conventional chemotherapy; HDC, high-dose chemotherapy; MO, microdonts; SR, short-rooted teeth; TA, tooth agenesis; TBI, total body irradiation; TFA, tooth formation anomaly.

### Dental examination

Personal oral plaster study models and orthopantomography were examined for tooth formation anomalies. The ratio between the root and crown lengths was calculated by orthopantomography. The longest root in multi-rooted molars, and buccal roots in the maxillary molars and premolars were used in the calculations. This study has excluded any consideration of third molars.

### Incidence of conventional chemotherapies

The tooth formation anomalies were evaluated by the Hölttä's Defect Index with simplifying the assessment of the ratio between the root and crown lengths (Hölttä *et al*. 2002, 2005a,b). The severity of tooth formation anomalies was determined by orthopantomography and personal study models according to the criteria: score 0 weighted on teeth having normal morphology, teeth not reliably observed on orthopantomography, developing teeth with an unclear final outcome at the age of the examination, and third molars; score 1 on short-rooted teeth of which the ratio between the root and crown lengths is lower than 1.6 considering the ratio of normal root development ≥1.6; score 2 weighted on microdonts; score 3 weighted on tooth agenesis. The numbers of short-rooted teeth, microdonts, and tooth agenesis were counted and the added weighted scores (1n + 2n + 3n) were analysed as the tooth formation anomaly scores for a participant. The incidence of tooth formation anomalies was determined by a formula: the respective number of tooth agenesis, microdonts, or short-rooted teeth divided by 28 (permanent tooth number except third molars).

### Statistical methods

The incidence ratio and scores of tooth formation anomalies were examined by the Kolmogorov–Smirnov test, correlation was by simple regression analysis, and differences between the groups were by unpaired two-tailed Student's *t*-test, and the non-parametric Mann–Whitney *U*-test. A *P*-value <0.05 was considered significant.

## Results

### Macroscopic examination of tooth formation anomalies

**Figure 1:**
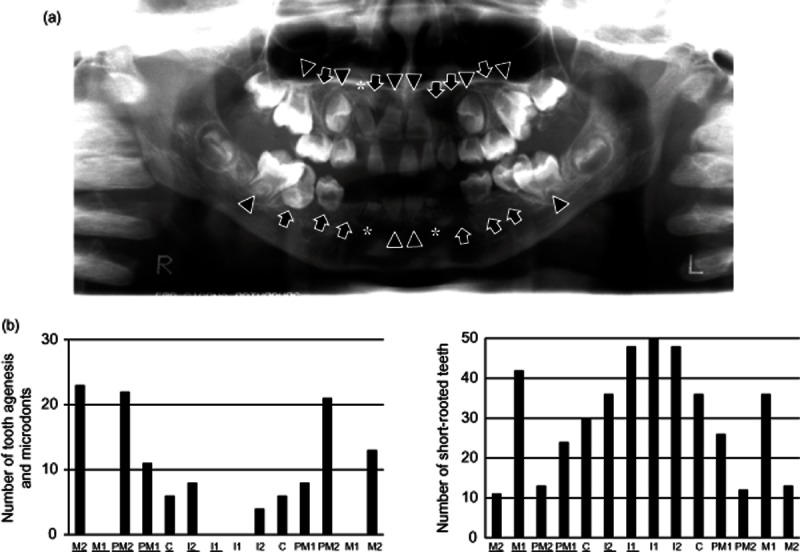
Emergence of chemotherapy-induced tooth formation anomalies. (a) Orthopantomograph of a case of chemotherapy-induced crown formation anomalies. The subject was treated with high-dose chemotherapy (HDC) at 1 year of age and the oral examination here was at 12 years of age. All permanent teeth except the third molars have anomalies: the bilateral upper lateral incisors, upper left canine, bilateral upper second premolars, bilateral lower canines, and bilateral lower first and second premolars display tooth agenesis (TA, arrows); the upper right canine and bilateral lower lateral incisors microdonts (MO, asterisks); teeth not subject to TA/MO short-rooted teeth (SR, arrowheads). (b) Permanent teeth with chemotherapy-induced anomalies. Central and lateral incisors, canines, first and second premolars, and first and second molars are expressed as I1 and I2, C, PM1 and PM2, and M1 and M2. Upper teeth are underlined. The highest incidence was in the second premolars and second molars with tooth agenesis or microdonts, and in the incisors with short-rooted teeth. Tooth agenesis or microdonts did not occur in the upper and lower first molars and central incisors.

The orthopantomograph in Figure 1 shows a case of chemotherapy-affected tooth formation anomalies in a 12-year-old subject who has undergone HDC at 1 year of age. All permanent teeth except the third molars display tooth formation anomalies: tooth agenesis in the incisors, canines, second premolars; microdonts in the upper canine, and lower lateral incisors; and short-rooted teeth in all other affected teeth. All subjects showed tooth agenesis/microdonts most in the second premolars and second molars, and short-rooted teeth emerged mostly in the incisors and first molars (Fig. 1). There was no tooth agenesis/microdonts in the first molars or in the central incisors.

### Incidence of tooth formation anomalies

There were tooth formation anomalies in 89.1% (41/46) of all subjects (Tables S2 and S3). The short-rooted teeth occurred in subjects who have undergone chemotherapy between 0.0 and 11.8 years of age, and the ratio of subjects with short-rooted teeth was highest for those with tooth agenesis/microdonts: 57.8% (15/26), 85.7% (12/14), and 100% (6/6) in the conventional chemotherapy, HDC, and HDC with TBI groups. None of tooth agenesis/microdonts was found in subjects who had undergone chemotherapy at 8 years of age and over. The ratio of subjects with tooth agenesis/microdonts was 66.7% (10/15) and 18.2% (2/11) in subjects administered conventional chemotherapy at <4 years and >4 years of age, respectively, while it was 100% (8/8) and 25% (3/12) in subjects administered HDC <4 years and >4 years of age. All of the incidences for tooth agenesis, microdonts and short-rooted teeth, and also tooth formation anomaly scores were higher in the HDC groups than in the conventional chemotherapy groups (Table 2).

### Correlation of the incidence of tooth formation anomalies to the age and duration of chemotherapy

**Figure 2:**
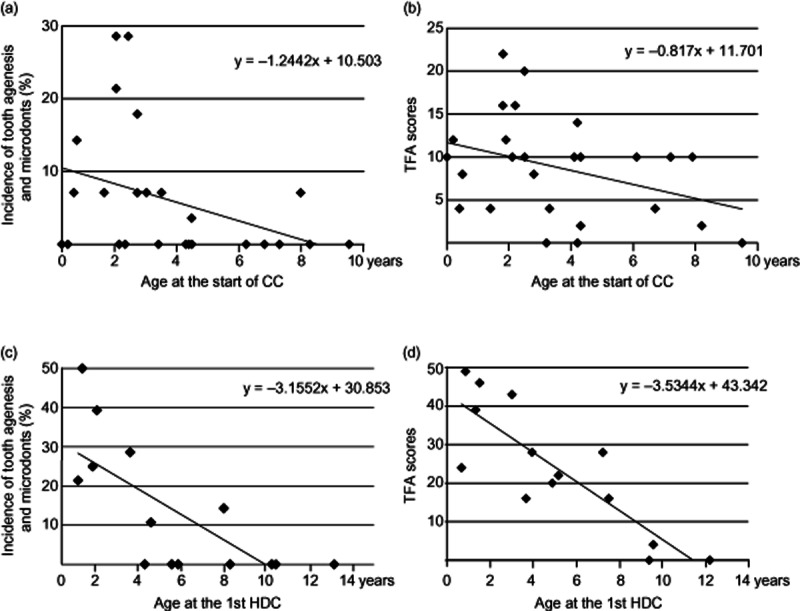
Co-relation of the incidence of tooth formation anomalies to the age at chemotherapy. (a) In the conventional chemotherapy (CC) group, the incidence of tooth agenesis or microdonts is not normally distributed or correlated to the age at the start of CC (y = −1.2442x + 10.503, R^2^ = 0.1335). (b) In the CC group, scores of tooth formation anomalies (TFA) are not normally distributed or correlated to the age at the start of CC (y = −0.817x + 11.701, R^2^ = 0.1299). (c) In the CC + high-dose chemotherapy without total body irradiation (HDC) group, the incidence of tooth agenesis or microdonts showed a normal distribution but no statistical correlation to the age at the 1st high-dose chemotherapy (y = −3.1552x + 30.853, R^2^ = 0.525). (d) In the HDC group, TFA scores showed both a normal distribution and statistical correlation to the age at the 1st high-dose chemotherapy (y = −3.5344x + 43.342, R^2^ = 0.7122).

**Figure 3:**
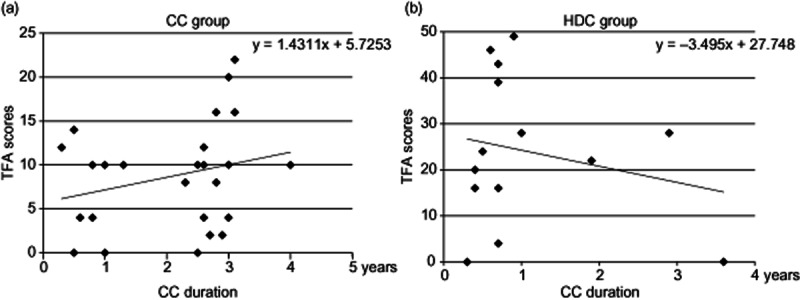
Co-relation of the incidence of tooth formation anomalies to the duration of conventional chemotherapy. (a) Scores of tooth formation anomalies (TFA) in the conventional chemotherapy (CC) group did not show a statistical correlation to the duration from the start to the end of CC (CC duration) (y = 1.4311x + 5.7253, R^2^ = 0.0674). (b) Scores of TFA in the CC + high-dose chemotherapy without total body irradiation (HDC) group did not show a correlation to CC duration (y = −3.495x + 27.748, R^2^ = 0.0465).

In the conventional chemotherapy group, the incidence of tooth agenesis/microdonts did not show a normal distribution or a significant correlation to the age at the start of conventional chemotherapy (R^2^ = 0.1335) (Fig. 2). Further, the tooth formation anomaly scores did not show a normal distribution or a significant correlation to the age at the start of conventional chemotherapy (R^2^ = 0.1299). The incidence of tooth agenesis/microdonts in the HDC group showed a normal distribution but did not show a significant correlation to the age at the 1st HDC (R^2^ = 0.525). The tooth formation anomaly scores in the HDC group showed both a normal distribution and a significant correlation to the age at the 1st HDC (R^2^ = 0.7122). The tooth formation anomaly scores in the conventional chemotherapy group did not show a correlation to the duration from the start to the end of conventional chemotherapy (conventional chemotherapy duration) (R^2^ = 0.0674) (Fig. 3). Further, tooth formation anomaly scores in the HDC group did not show a significant correlation to the conventional chemotherapy duration (R^2^ = 0.0465).

### Comparison of the tooth formation anomalies between the conventional chemotherapy and HDC groups

**Figure 4:**
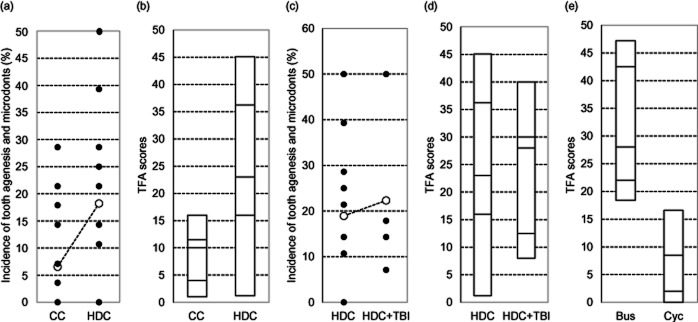
Comparison of tooth formation anomalies among subjects with conventional chemotherapy or high-dose chemotherapy with/without total body irradiation. (a) Comparison of the incidence of tooth agenesis and microdonts between the conventional chemotherapy (CC) and CC + high-dose chemotherapy without total body irradiation (HDC) groups. In subjects who started CC or were administered the 1st high-dose chemotherapy below eight years of age, the incidence of tooth agenesis and microdonts was significantly higher (*P* < 0.05) in the HDC group (*n* = 10) than in the CC group (*n* = 24). Open dots express medians. (b) Comparison of scores of tooth formation anomalies (TFA) between the CC and HDC groups. TFA scores were significantly higher in the HDC group (*n* = 14) than in the CC group (*n* = 26) (*P* < 0.01). Lines in bars express TFA scores at the 10th, 25th, 50th (median), 75th, and 90th percentiles. (c) Comparison of the incidence of tooth agenesis and microdonts between the HDC and HDC + total body irradiation (TBI) groups. In subjects who were administered the 1st high-dose chemotherapy below 8 years of age, the incidence of tooth agenesis and microdonts was not significantly different between the HDC (*n* = 10) and HDC with TBI (*n* = 4) groups. Open dots express median. (d) Comparison of TFA scores between the HDC and HDC + TBI groups. TFA scores were not significantly different between the HDC (*n* = 14) and HDC with TBI (*n* = 6) groups. Lines in the bars express TFA scores at the 10th, 25th, 50th (median), 75th, and 90th percentiles. (e) Comparison of TFA scores between the busulfan and cyclophosphamide-treated subjects. In the HDC group, TFA scores were significantly higher in the busulfan (Bus)-administrated subjects (*n* = 7) than in the cyclophosphamide (Cyc)-administrated subjects (*n* = 4) (*P* < 0.05). Lines in bars express TFA scores at the 10th, 25th, 50th (median), 75th, and 90th percentiles.

The average incidences of tooth agenesis/microdonts and the tooth formation anomaly scores for the conventional chemotherapy and the HDC groups were compared (Fig. 4). In subjects who started conventional chemotherapy or were administered the 1st HDC at less than eight years of age, the incidence of tooth agenesis/microdonts was significantly higher (*P* < 0.05) in the HDC group (*n* = 10) than in the conventional chemotherapy group (*n* = 24), further in subjects who started conventional chemotherapy or were administered the 1st HDC at less than four years of age, the incidence of tooth agenesis/microdonts in the HDC group (*n* = 5) was significant at the 1% level when compared with the conventional chemotherapy group (*n* = 15) (data not shown). The tooth formation anomaly scores of all subjects are significantly higher in the HDC group than in the conventional chemotherapy group (*P* < 0.01).

### Comparison of tooth formation anomalies between HDC and HDC + TBI

In subjects who were administered the 1st HDC at less than eight years of age, the incidence of tooth agenesis/microdonts was not significantly different for the HDC with TBI (*n* = 4) and without TBI (*n* = 10) groups, and the tooth formation anomaly scores of all subjects was not significantly different for the HDC without TBI (*n* = 6) and with TBI (*n* = 14) groups (Fig. 4).

### Comparison of the tooth formation anomalies between busulfan and cyclophosphamide

In subjects treated by HDC, the tooth formation anomaly scores were significantly higher in the busulfan-administrated subjects (*n* = 7) than in the cyclophosphamide-administrated subjects (*n* = 4) (*P* < 0.05) (Fig. 4).

## Discussion

### Types of teeth with chemotherapy-induced anomalies

The ratios of subjects with at least one tooth formation anomaly were more than 85% in all study groups. Tooth germ formation of incisors, first premolars, and first molars are completed by birth, while tooth germ formation of second premolars and second molars begins at 7–9 months after birth. The age at the start of calcification is within 1 year after birth for incisors and first molars, while it is 1–3 years of age for premolars and second molars. The crowns are completed at 2–8 years of age and the roots are completed by the age of 16 (Sterrett *et al*. 1999; Endo *et al*. 2004, 2006; Fujita *et al*. 2009; Abe *et al*. 2010). Further, it is generally accepted that in the healthy Japanese population the prevalence of tooth agenesis/microdonts, other than third molars, is 0.0% to 2.4%. Therefore, it is likely that persons under 8 years of age undergoing chemotherapy are at risk for tooth agenesis/microdonts and persons under 16 are at risk for short-rooted teeth. This study has excluded any consideration of third molars because the tooth germ formation and calcification starts at the ages of 3–4 and 7–10 respectively, and further the generic rate of congenitally missing third molars is about 35% in Japan (Sterrett *et al*. 1999; Endo *et al*. 2004, 2006; Fujita *et al*. 2009; Abe *et al*. 2010). The incidence of short-rooted teeth was higher than tooth agenesis/microdonts in all study groups (Table 2). It would be due to the completion of permanent tooth roots other than third molars by 16 years of age. It is clear that the QOL is poor in subjects with multiple crown formation anomalies and that it is more meaningful not to count microdonts alone because of the similar disfunction between microdonts and tooth agenesis (Fig. 1). The highest rate of occurrence was in the second premolars and second molars for tooth agenesis/microdonts, and in incisors and first molars for short-rooted teeth while in the key teeth with the earliest age of calcification, tooth agenesis/microdonts were absent in first molars and central incisors, and present in some canines. Considering these, the occurrence of crown formation anomalies is predicted in the second premolars and second molars by the chemotherapies for paediatric cancers.

### Age at chemotherapy resulting in tooth formation anomalies

The ratio of subjects with tooth agenesis/microdonts was significantly higher in subjects treated at under 4 years of age than in subjects treated at over 4 years of age in all study groups. In the conventional chemotherapy group, the incidence of tooth agenesis/microdonts and tooth formation anomaly scores did not show a normal distribution (Fig. 2). In the HDC group, the incidence of tooth agenesis/microdonts did not show a correlation with age for HDC. These suggest that undergoing conventional chemotherapy or HDC below 4 years of age introduces a high-risk factor for dentition leading to frequent tooth agenesis/microdonts while it is rarely a risk factor at over 4 years of age. In the conventional chemotherapy group, the tooth formation anomaly scores did not show a normal distribution or significant correlation to the age at the start of conventional chemotherapy, whereas in the HDC group, the tooth formation anomaly scores showed a normal distribution and a significant correlation to the age at HDC (Fig. 2). These suggest that the risk to cause tooth formation anomalies by conventional chemotherapy increases with HDC. Further, neither the tooth agenesis/microdonts incidence nor tooth formation anomaly scores correlate to the conventional chemotherapy duration in the conventional chemotherapy or HDC groups (Fig. 3). These suggest that the duration and protocols of conventional chemotherapy is not an important risk factor to cause the tooth formation anomalies.

### Risk factors for tooth formation anomalies in paediatric cancer treatment

The incidence of tooth agenesis/microdonts in subjects treated by chemotherapy at below 8 years of age and the tooth formation anomaly scores were higher in the HDC group than in the conventional chemotherapy group (Fig. 4), suggesting that the HDC increases the risk of tooth formation anomalies. Further, the chemotherapy at below 4 years of age becomes a higher risk of the occurrence tooth agenesis/microdonts. The incidence of tooth agenesis/microdonts in subjects with HDC at under 8 years of age and the tooth formation anomaly scores were not significantly different for the HDC without TBI and with TBI groups (Fig. 4). These findings suggest that the HDC may mask the emergence of tooth formation anomalies due to the radiation injuries caused by TBI. Further, it has been reported that cyclophosphamide in HDC acts as a high-risk factor of tooth formation anomalies (Kaste *et al*. 2009; Hsieh *et al*. 2011). In the subjects who have undergone HDC, the tooth formation anomaly scores were significantly higher in busulfan-administered subjects than in cyclophosphamide-administered subjects (Fig. 4). These suggesting that the risk to cause tooth formation anomalies with busulfan administration is higher than with cyclophosphamide, and that the influence to tooth development is different among alkylators.

In conclusion, the anti-cancer chemotherapy under 8 years of age becomes a risk factor in tooth agenesis or microdonts in second premolars and second molars, significantly increasing by the HDC treatment. However, the chemotherapy to children at over 4 years of age has little risk of inducing serious crown formation anomalies. The protocols of conventional chemotherapy are not an important risk factor to cause tooth formation anomalies but the risk may be higher with busulfan than with cyclophosphamide in the HDC. The TBI does not increase the risk of tooth formation anomalies in the HDC-administered subjects.

## SUPPORTING INFORMATION

**Table S1.** Duration of conventional chemotherapy and age at high-dose chemotherapy

**Table S2.** Numbers of permanent teeth with formation anomalies in CC

**Table S3.** Numbers of permanent teeth with formation anomalies in the high-dose chemotherapy
