# Supplementary material for: Risk factors to cause tooth formation anomalies in chemotherapy of paediatric cancers
Source: Eur J Cancer Care (Engl). 2013 Jan 21;22(3):353–60. doi: 10.1111/ecc.12038 (PMC3655612; doi:10.1111/ecc.12038)
Supplement: Supplementary file 1 [file ecc0022-0353-SD1.docx]

| Supplementary table I. Duration of conventional chemotherapy and age at high-dose chemotherapy. | | | | | | | |
| --- | --- | --- | --- | --- | --- | --- | --- |
| Study group | | Age at oral examination (yrs) | Age of onset (yrs) | Age at the start of CC (yrs) | Age at the end of CC (yrs) | CC duration (yrs) | Age at the 1st HDC (yrs) |
| CC  (n=26) | Ra | 7.0-32.2 | 0.0-9.5 | 0.0-9.5 | 0.4-15.7 | 0.3-4.0 |  |
|  | Av | 18.5 | 3.6 | 3.6 | 6.1 | 2.1 |  |
| CC+HDC^a^ (n=20) | Ra | 10.7-32.0 | 0.1-9.8 | 0.1-10.0 | 0.8-12.5 | 0.3-3.6 | 0.8-13.1 |
|  | Av | 16.7 | 3.9 | 4.0 | 5.5 | 1.3 | 5.7 |
| CC+HDC without TBI  (n=14) | Ra | 10.7-32 | 0.1-9.8 | 0.1-10.0 | 0.8-10.4 | 0.3-3.6 | 0.8-13.1 |
|  | Av | 17.0 | 4.0 | 4.0 | 5.1 | 1.1 | 5.5 |
| CC+HDC with TBI (n=6) | Ra | 12.3-19.2 | 1.1-6.8 | 1.1-6.8 | 1.8-12.5 | 0.4-3.5 | 1.8-11.2 |
|  | Av | 16.0 | 3.9 | 4.0 | 6.5 | 1.7 | 6.1 |
| Total^b^  (n=46) | Ra | 7.0-32.2 | 0.0-9.8 | 0.0-10.0 | 0.4-15.7 | 0.3-4.0 |  |
|  | Av | 17.7 | 3.7 | 3.8 | 5.8 | 1.8 |  |
| CC: conventional chemotherapy; HDC: high-dose chemotherapy; TBI: total body irradiation; Ra: range; Av: average. ^a^Total subjects treated by CC+HDC with or without TBI. ^b^Total subjects treated by CC or CC+HDC. | | | | | | | |
