# Supplementary material for: Risk factors to cause tooth formation anomalies in chemotherapy of paediatric cancers
Source: Eur J Cancer Care (Engl). 2013 Jan 21;22(3):353–60. doi: 10.1111/ecc.12038 (PMC3655612; doi:10.1111/ecc.12038)
Supplement: Supplementary file 2 [file ecc0022-0353-SD2.docx]

| Supplementary table II. Numbers of permanent teeth with formation anomalies in CC. | | | | | | | | | |
| --- | --- | --- | --- | --- | --- | --- | --- | --- | --- |
| Case No./ gender | Diagnosis | Age at the start of CC (yrs) | Age at the 1st HDC (yrs) | Age at oral examination (yrs) | CC duration (yrs) | Numbers of TFA | | | TFA scores |
|  |  |  |  |  |  | TA | MO | SR |  |
|  |  |  |  |  |  |  |  |  |  |
| CC (n=26) | | | | | | | | | |
| 1/M | AML | 0.0 |  | 13.9 | 0.8 | 0 | 0 | 10 | 10 |
| 2/M | NBL | 0.2 |  | 11.4 | 0.3 | 0 | 0 | 12 | 12 |
| 3/M | ALL | 0.4 |  | 14.3 | 0.6 | 0 | 2 | 0 | 4 |
| 4/F | ALL | 0.5 |  | 21.8 | 2.3 | 0 | 4 | 0 | 8 |
| 5/M | HBL | 1.4 |  | 13.8 | 0.8 | 0 | 2 | 0 | 4 |
| 6/F | ALL | 1.8 |  | 26.6 | 2.8 | 0 | 8 | 0 | 16 |
| 7/M | ALL | 1.8 |  | 21.9 | 3.1 | 0 | 6 | 10 | 22 |
| 8/F | ALL | 1.9 |  | 21.4 | 2.6 | 0 | 0 | 12 | 12 |
| 9/F | ALL | 2.1 |  | 20.0 | 2.5 | 0 | 0 | 10 | 10 |
| 10/F | ALL | 2.2 |  | 24.0 | 3.1 | 0 | 8 | 0 | 16 |
| 11/M | AML | 2.5 |  | 7.0 | 1.0 | 0 | 5 | 0 | 10 |
| 12/F | ALL | 2.5 |  | 15.7 | 3.0 | 0 | 2 | 16 | 20 |
| 13/M | ALL | 2.8 |  | 14.2 | 2.8 | 0 | 2 | 4 | 8 |
| 14/F | ACC | 3.2 |  | 7.9 | 1.0 | 0 | 0 | 0 | 0 |
| 15/F | ALL | 3.3 |  | 17.6 | 2.6 | 0 | 2 | 0 | 4 |
| 16/F | ALL | 4.1 |  | 8.8 | 3.0 | 0 | 0 | 10 | 10 |
| 17/F | ALL | 4.2 |  | 22.1 | 2.5 | 0 | 0 | 0 | 0 |
| 18/F | MBL | 4.2 |  | 9.3 | 0.5 | 0 | 0 | 14 | 14 |
| 19/F | ALL | 4.3 |  | 28.8 | 2.7 | 0 | 1 | 0 | 2 |
| 20/M | ML | 4.3 |  | 26.8 | 4.1 | 0 | 0 | 10 | 10 |
| 21/M | ALL | 6.1 |  | 9.1 | 1.3 | 0 | 0 | 10 | 10 |
| 22/M | ALL | 6.7 |  | 32.2 | 3.0 | 0 | 0 | 4 | 4 |
| 23/M | ALL | 7.2 |  | 23.2 | 3.0 | 0 | 0 | 10 | 10 |
| 24/F | ALL | 7.9 |  | 23.9 | 2.6 | 2 | 0 | 4 | 10 |
| 25/M | ALL | 8.2 |  | 18.3 | 2.9 | 0 | 0 | 2 | 2 |
| 26/F | ALL | 9.5 |  | 26.6 | 0.5 | 0 | 0 | 0 | 0 |
