# Supplementary material for: Risk factors to cause tooth formation anomalies in chemotherapy of paediatric cancers
Source: Eur J Cancer Care (Engl). 2013 Jan 21;22(3):353–60. doi: 10.1111/ecc.12038 (PMC3655612; doi:10.1111/ecc.12038)
Supplement: Supplementary file 3 [file ecc0022-0353-SD3.docx]

| Supplementary table III. Numbers of permanent teeth with formation anomalies in the high-dose chemotherapy. | | | | | | | | | |
| --- | --- | --- | --- | --- | --- | --- | --- | --- | --- |
| Case No./ gender | Diagnosis | Age at the start of CC (yrs) | Age at the 1st HDC (yrs) | Age at oral examination (yrs) | CC duration (yrs) | Numbers of TFA | | | TFA scores |
|  |  |  |  |  |  | TA | MO | SR |  |
|  |  |  |  |  |  |  |  |  |  |
| CC+HDC without TBI (n=14) | | | | | | | | | |
| 1/M | AML | 0.3 | 0.8 | 13.5 | 0.5 | 2 | 4 | 10 | 24 |
| 2/M | PNET | 0.1 | 1.0 | 12.0 | 0.9 | 11 | 3 | 10 | 49 |
| 3/M | SB | 1.1 | 1.5 | 17.6 | 0.7 | 7 | 0 | 18 | 39 |
| 4/M | AML | 1.1 | 1.7 | 17.8 | 0.6 | 7 | 4 | 17 | 46 |
| 5/F | PNET | 2.5 | 3.3 | 15.8 | 0.7 | 7 | 1 | 20 | 43 |
| 6/F | AML | 3.6 | 4.0 | 21.8 | 0.4 | 0 | 0 | 16 | 16 |
| 7/M | HBL | 1.4 | 4.3 | 10.7 | 1.0 | 0 | 3 | 22 | 28 |
| 8/M | AML | 4.9 | 5.3 | 19.9 | 0.4 | 0 | 0 | 20 | 20 |
| 9/M | WT | 3.8 | 5.6 | 11.1 | 1.9 | 0 | 0 | 22 | 22 |
| 10/F | ALL | 4.3 | 7.8 | 22.8 | 2.9 | 0 | 4 | 20 | 28 |
| 11/M | MBL | 7.4 | 8.1 | 16.9 | 0.7 | 0 | 0 | 16 | 16 |
| 12/M | MBL | 10.0 | 10.2 | 11.8 | 0.6 | 0 | 0 | 0 | 0 |
| 13/M | MBL | 9.8 | 10.3 | 14.1 | 0.7 | 0 | 0 | 4 | 4 |
| 14/M | ALL | 5.3 | 13.1 | 32.0 | 3.6 | 0 | 0 | 0 | 0 |
| CC+HDC with TBI (n=6) | | | | | | | | | |
| 1/M | ALL | 1.1 | 1.8 | 15.1 | 0.7 | 8 | 6 | 14 | 50 |
| 2/M | ALL | 2.3 | 2.8 | 12.3 | 0.4 | 2 | 2 | 20 | 30 |
| 3/M | ALL | 2.1 | 2.9 | 15.5 | 1.0 | 0 | 5 | 20 | 30 |
| 4/F | NBL | 6.8 | 7.5 | 19.2 | 1.2 | 0 | 2 | 22 | 26 |
| 5/M | ALL | 5.9 | 10.8 | 15.8 | 3.4 | 0 | 0 | 8 | 8 |
| 6/F | ALL | 5.5 | 11.8 | 18.1 | 3.5 | 0 | 0 | 8 | 8 |
| CC: conventional chemotherapy; HDC: high-dose chemotherapy; TBI: total body irradiation; TFA: tooth formation anomalies; TA: tooth agenesis; MO: microdonts; SR: short-rooted teeth. | | | | | | | | | |
